# Indium-111-labelled octreotide scintigraphy in the diagnosis and management of non-iodine avid metastatic carcinoma of the thyroid

**DOI:** 10.1038/sj.bjc.6601072

**Published:** 2003-07-15

**Authors:** J A Christian, G J R Cook, C Harmer

**Affiliations:** 1 Thyroid Unit, Royal Marsden Hospital, Surrey, UK

**Keywords:** octreotide, Hurthle cell carcinoma, somatostatin receptor, radioiodine

## Abstract

Treatment of differentiated thyroid cancer is a success of modern medicine with the use of radioiodine (^131^I). However, a significant proportion of thyroid cancers may be non-iodine avid. Thyroid tumours are known to express somatostatin receptors. Octreotide, an analogue of somatostatin, can be combined with a radioactive isotope, such as ^111^In-DTPA^0^ to visualise tumours with high concentrations of somatostatin receptors. We assessed 18 patients with histologically proven metastatic or locally recurrent non-iodine avid thyroid carcinoma to determine the usefulness of ^111^In-DTPA^0^ octreotide scintigraphy compared to conventional radiology in diagnosing sites of metastasis. The diagnosis of metastatic disease was made using conventional radiology and all had prospective scintigraphy using ^111^In-DTPA^0^octreotide. Of the 18 patients, 14 had octreotide-positive scans. In eight, the octreotide scans identified the same sites of metastases as conventional radiology, that is, were concordant. In nine patients, conventional radiology showed more extensive disease than revealed on the octreotide scans. In one patient with widespread bone metastases, octreotide gave a more detailed assessment of metastatic disease than conventional radiology. These data indicate that ^111^In-DTPA^0^octreotide imaging for patients with non-iodine avid carcinoma of the thyroid may be a useful diagnostic and staging tool. One patient with Hurthle cell carcinoma metastatic to bone and a positive octreotide scan has been treated with ^90^yttrium-labelled octreotide.

Treatment of differentiated thyroid cancer is a success of modern medicine with the use of radioiodine (^131^I) for postoperative ablation of residual normal thyroid and for eradication of metastatic disease (Vini and Harmer, 2000). Follicular carcinoma accounts for approximately 20% of all thyroid cancer and is well known for its ability to concentrate iodine. However, the Hurthle cell carcinoma (HCC) variant, which represents approximately 3% of all differentiated thyroid cancers is rarely, if ever, iodine avid (Vini *et al*, 1998). It is frequently bilateral or multifocal within the thyroid gland and often presents with local invasion (Grossman and Clark, 1997). Hurthle cell carcinoma is associated with a high rate of locoregional recurrence and significant mortality. Nodal metastases and extrathyroidal extension predict a worse outcome (Stojadinovic *et al*, 2001). Survival analysis and disease-free intervals in one series demonstrated increasing tumour aggressiveness to be papillary, mixed, follicular and HCC, respectively (Samaan *et al*, 1983). Apart from HCC, only two thirds of patients with differentiated thyroid carcinoma will concentrate iodine in their metastases and, during the course of the disease, uptake will disappear in a further significant proportion (Schlumberger *et al*, 1996). Further surgery and external beam radiotherapy are the mainstay of management for these patients when disease is localised. For metastatic disease, local palliative radiotherapy may also be valuable, but response to chemotherapeutic agents is usually poor.

Somatostatin is a short-acting regulatory peptide hormone containing a 14 amino-acid chain that has a predominantly inhibitory role in hormone release within the gastrointestinal tract and a neurotransmitter/modulatory role in the central nervous system. However, receptors for somatostatin, which are G-protein-coupled receptors, occur in multiple sites throughout the body such as thyroid C cells, lymphocytes, pancreas and the somatotroph cells of the anterior pituitary. Five human somatostatin receptor subtypes (sstrs) have been identified (Bell *et al*, 1995), which interact with different G-proteins to mediate effects via inhibition of adenylate cyclase activity (Rens-Domiano *et al*, 1992; Law *et al*, 1993). When octreotide, a synthetic analogue of somatostatin with a considerably longer half-life, is combined with a radioactive isotope such as indium-111 (emits gamma photons of energies 172 and 245 keV and Auger electrons with tissue penetration 0.02–500 μm), peptide receptor imaging can be performed to visualise tumours with a high concentration of somatostatin receptors (Wilson *et al*, 1998). Multiple somatostatin receptor subtypes are known to be present in medullary thyroid cancer, but actually the majority of thyroid tumours regularly express most, if not all, of the somatostatin receptor subtypes (Forssell-Aronsson *et al*, 2000). Studies indicate the existence of two groups of receptors – sstr1/sstr4 with virtually no or very low affinity and sstr2/sstr3/sstr5 with intermediate to high affinity for the somatostatin analogues, such as octreotide and lanreotide (Bruns *et al*, 1995). The density of the sstrs will affect radioisotope imaging and targeted therapy.

In this study, patients known to have non-iodine avid metastatic thyroid carcinoma were assessed with ^111^In-DTPA^0^octreotide scintigraphy (DTPA=diethylenetriaminepentaacetic acid) and this was compared with conventional radiology. The objectives were to evaluate ^111^In-DTPA^0^octreotide scintigraphy as a diagnostic tool and to consider its potential for therapy.

## METHODS AND MATERIALS

In total, 18 patients at the Royal Marsden Hospital with non-iodine avid metastatic or locally recurrent thyroid carcinoma were identified from the Thyroid Unit computer database of over 2000 patients. All had histologically confirmed diagnosis of thyroid cancer, with initial treatment usually comprising total thyroidectomy±selective neck dissection. This was followed by TSH suppression with thyroxine replacement (with exception of the patient with medullary thyroid carcinoma (MTC) where thyroxine replacement was not TSH suppressive). A diagnosis of metastatic disease was made using conventional radiology: plain X-ray, ultrasound, ^99m^Tc bone scintigraphy, computerised tomography (CT) or magnetic resonance imaging (MRI). Metastatic disease found on conventional radiology was confirmed using serial measurements of serum thyroglobulin (or calcitonin in the case of MTC).

Patients underwent diagnostic whole-body scanning with radioiodine (^131^I) and prospective scintigraphy using ^111^In-DTPA^0^octreotide (OctreoScan®, Mallinckrodt Medical B.V.). Planar and SPECT images were obtained 24 h after injection of 111 MBq ^111^In-DTPA^0^octreotide. Occasionally, these were repeated at 48 h if there was excessive bowel uptake at 24 h. All imaging was reported by experienced radiologists and nuclear medicine physicians; abnormalities had to be beyond reasonable doubt or demonstrate progression from previous scans before being described as metastasis. Sites of metastases were correlated with results from conventional imaging. When the octreotide scan demonstrated each site of metastasis found with conventional radiology, the imaging was judged ‘concordant’. If either conventional radiology or the octreotide scan demonstrated more extensive metastatic disease than the other, then it was designated ‘better’.

## RESULTS

In total, 18 patients with histologically proven metastatic thyroid cancer underwent prospective scintigraphy with ^111^In-DTPA^0^octreotide as shown in Table 1

**Table 1:** Comparison of conventional radiology and ^111^In-DTPA^0^octreotide scintigraphy in 18 patients with non-iodine avid metastatic thyroid carcinoma

	**Histology**	**Site of metastases**	**Octreotide**	**Concordance**
1	Hurthle	Bone, lymph nodes, liver, adrenal gland	Negative	Conventional radiology better
2	Hurthle	Lung, hilar lymph nodes, brain	Positive	Conventional radiology better
3	Hurthle	Lung, cervical lymph nodes	Positive	Concordant
4	Hurthle	Skull base	Negative	Conventional radiology better
5	Hurthle	Residual thyroid disease	Positive	Concordant
6	Hurthle	Lung, mediastinal lymph nodes	Positive	Conventional radiology better
7	Hurthle	Bone	Positive	Octreotide better
8	Hurthle	Recurrent soft tissue neck disease	Positive	Concordant
9	Hurthle	Brain, cervical and mediastinal lymph nodes	Positive	Conventional radiology better
10	Hurthle	Sternum	Positive	Concordant
11	Hurthle	Mediastinal lymph node mass	Positive	Concordant
12	Hurthle	Lung	Positive	Concordant
13	Papillary	Bone, lung	Positive	Conventional radiology better
14	Papillary	Liver, lung, mediastinal and hilar lymph nodes	Positive	Conventional radiology better
15	Papillary	Lung	Negative	Conventional radiology better
16	Papillary	Paratracheal lymphnode mass	Positive	Concordant
17	Medullary	Residual soft tissue neck disease	Positive	Concordant
18	Follicular	Lung	Negative	Conventional radiology better

LNs=lymph nodes, CR=conventional radiology.

. There were 12 patients with HCC, four with papillary carcinoma, one with follicular carcinoma and one with MTC. In all, 11 had previously undergone ^131^I scintigraphy, which had been negative. Of the remaining seven patients, six had HCC and one had MTC–both characteristically non-iodine avid.

**Figure 1:**
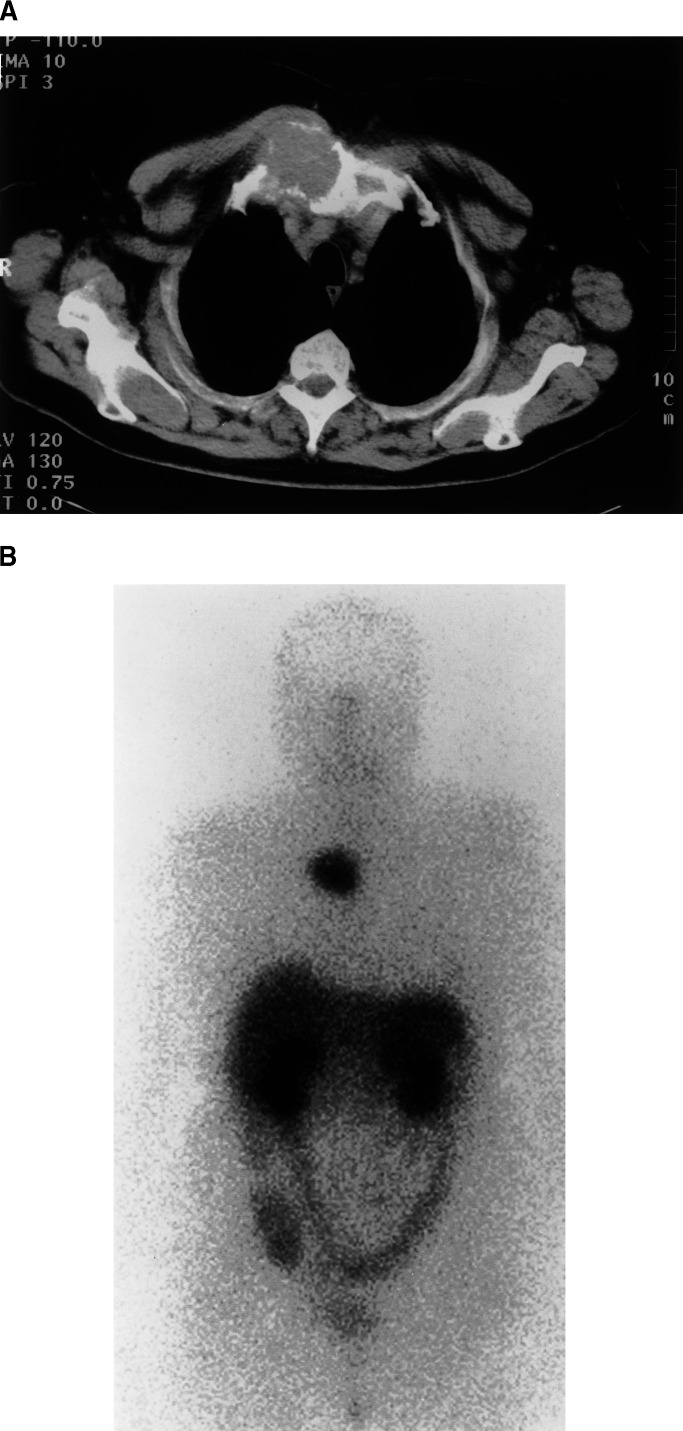
Comparative images of a patient with an isolated sternal metastasis secondary to a Hurthle cell thyroid primary tumour. Image (**A**) is cross-sectional CT slice through the deposit. Image (**B**) is the ^111^In-DTPA^0^octreotide scan showing intense uptake at the site of the deposit.

Of 18 patients, 14 (78%) had ^111^In-DTPA^0^octreotide-positive scans that demonstrated abnormalities indicative of metastases. In eight (44%), there was an excellent correlation (concordance) between disease found on conventional radiology and that found with octreotide imaging (Figure 1

). In five patients (28%) conventional radiology showed that disease was more widespread than indicated by octreotide imaging although this was positive in some areas. Four patients (22%) with visible metastatic disease on conventional radiology had negative octreotide scans. In one patient (6%) with bone metastases only, octreotide scintigraphy demonstrated more extensive involvement compared with conventional radiology including ^99m^Tc bone scintigraphy. See Table 1.

## DISCUSSION

Our results show that ^111^In-DTPA^0^octreotide imaging may be useful for both staging and monitoring of disease in patients with non-iodine avid thyroid carcinoma. Of 18 patients with metastatic disease, 14 showed positive uptake with ^111^In-DTPA^0^octreotide and in eight of these patients, it was as good as or better than conventional imaging. Imaging with octreotide does not provide an alternative to conventional radiology in the staging of non-iodine avid thyroid cancer, but may provide additional useful information, particularly in the case of widespread bone metastases. It is well established that the optimum method of demonstrating skeletal metastases in differentiated thyroid cancer is by ^131^I scintigraphy, rather than ^99m^Tc bone scan, due to their lytic nature. It is perhaps not surprising that, if these bone metastases also express somatostatin receptors, octreotide scintigraphy may prove superior to ^99m^Tc bone scintigraphy.

Our results compare favourably with other studies that have assessed octreotide scintigraphy in patients with HCC. Tisell *et al* reported a series of 10 patients with HCC, eight of whom had disease localised to the thyroid and this was shown preoperatively as distinct areas of increased uptake of radionuclide. Two further patients with metastatic HCC had metastases visualised by octreotide scintigraphy (Tisell *et al*, 1999). Valli *et al* (1999) used somatostatin receptor imaging in an attempt to detect metastases in patients with previously treated non-iodine avid thyroid cancer and an elevated thyroglobulin but was of no benefit over conventional imaging.

In a study by Gorges *et al* (1999) eight of nine patients with metastatic HCC showed ^111^In-pentetreotide accumulation of various intensity, while ^131^I scans were negative except for one patient. The octreotide scans were compared to FDG-PET scans and found to be slightly inferior. A major advantage of scintigraphy using octreotide compared to radioiodine is that patients do not have to withdraw thyroid hormone. This was emphasised by Haslinghuis *et al* (2001) in a series of 25 patients with differentiated thyroid cancer, who also showed that 75% patients with lesions that did not concentrate radioiodine did show uptake of octreotide. Gulec *et al* (1998) described three patients with HCC and found all to have positive octreotide scintigraphy. Different methods of assessing distant metastases in HCC have also been described, such as lung deposits demonstrated with ^131^I labelled anti-carcinoembryonic antigen (CEA) monoclonal antibody (Abdel-Nabi *et al*, 1985) and the incidental finding of HCC in a neck mass on an FDG-PET during staging for malignant melanoma (Wiesner *et al*, 1999).

As useful as peptide receptor scintigraphy is with ^111^In-DTPA^0^octreotide in diagnosis and monitoring of metastatic non-iodine avid thyroid cancer, the major interest lies in its potential for peptide receptor targeted therapy. When the radiopharmaceutical is concentrated within cells, the radioactivity remains in close proximity to the nuclear DNA, making the radiotoxicity of the Auger electrons high as it is within their particle range. One patient from our institution with bone metastases only and a strongly positive octreotide scan has undergone treatment with the high-energy beta-emitting ^90^Y DOTATOC (^90^Y DOTA,_D-_Phe^1^,Tyr^3^) octreotide (DOTA=tetraazacyclododecanetetraacetic acid) and she has experienced excellent palliation with diminution of pain. Krenning has suggested that, depending on the homogeneity of distribution of tumour cells expressing peptide receptors and the size of tumour, beta-emitting radionuclides, such as ^90^Y labelled to DOTA-chelated peptides, may be more effective than ^111^In for therapy (Krenning *et al*, 2000). Waldherr *et al* have published a series of 20 patients with progressive thyroid carcinoma who were refractory to treatment. Although none were reported as having HCC, all had either a positive ^111^In-DOTATOC scintiscan or ^111^In-DTPA^0^octreotide scintiscan. They were treated with 1700–7400 MBq m^−2 90^Y-DOTATOC, which was well tolerated. Stable disease was reported in 35% and progressive disease in 65%; none achieved partial or complete response (Waldherr *et al*, 2001). Therapy with ^111^In-DTPA^0^octreotide has been reported by Valkema *et al* (2002) in a phase I trial. In all, 50 patients with malignant somatostatin receptor positive tumours were treated, including six MTC, four papillary and one follicular thyroid cancers. Of 40 evaluable patients, there was therapeutic benefit (stabilisation or regression in tumour) seen in 21 (52.5%). Cumulative doses ranged from 20 to 160 GBq and toxicity was generally only mild bone marrow suppression. However, three out of six patients who received >100 GBq developed a myelodysplastic syndrome or leukaemia and 100 GBq was therefore set as the maximum tolerable dose. No significant renal toxicity was noted.

Preliminary results of MAURITIUS (Multicenter Analysis of a Universal Receptor Imaging and Treatment Initiative, a European Study) have recently been published (Virgolini *et al*, 2002). Using cumulative treatment doses up to 7.1 GBq, ^90^Y-DOTA–lanreotide was used to treat 25 patients with thyroid cancer refractory to conventional treatment; 56% demonstrated either regression response (>25% reduction in tumour size) or stable disease after treatment with one to four doses. No severe acute or chronic haematologic toxicity or change in renal or liver function parameters was found. The authors recommended careful consideration of the type of radiotracer for each patient. Whole-body dosimetry was recommended to predict absorbed doses to tumours, kidney and bone marrow.

Recently, attempts have been made to induce re-differentiation in non-iodine avid thyroid tumours using retinoids so as to increase radioiodine uptake. Simon *et al* reported a series of 50 patients with inoperable, non-iodine avid advanced thyroid cancer treated with 13-*cis*-retinoic acid. In all, 13 showed a clear increase in radioiodine uptake and eight, a mild increase. Thyroglobulin levels were unchanged or decreased in 20 patients (Simon *et al*, 2002). However, experience at our institution, using retinoic acid to increase radioiodine uptake in a similar patient group (Short *et al*, 2001) has demonstrated no worthwhile benefit. It may still prove that retinoic acid not only increases radioiodine uptake, but also the uptake of ^111^In-DTPA^0^octreotide.

## CONCLUSIONS

Our study shows that imaging with ^111^In-DTPA^0^octreotide may be useful both in the staging and monitoring of patients with non-iodine avid carcinoma of the thyroid. Tumour that has been identified may be surgically resectable and could result in prolongation of survival (Vini and Harmer, 2002). For unresectable disease, diagnostic scan positive patients may progress to somatostatin receptor-targeted therapy. It remains to be seen whether or not receptor-targeted therapy will prove to be a valuable treatment, but patients with non-iodine avid metastatic thyroid cancer currently face inexorable tumour progression with no worthwhile alternative therapy.
